# Method for Extraction and Evaluation of *Heliocarpus popayanensis* and *Triumfetta bogotensis* as Natural Coagulants for the Treatment of Wastewater

**DOI:** 10.3390/mps6060105

**Published:** 2023-11-02

**Authors:** Yeison Alberto Garcés-Gómez, Sebastián Isaac Pacheco-Gonzalez

**Affiliations:** Faculty of Engineering and Architecture, Catholic University of Manizales, Manizales 170001, Colombia; spacheco@ucm.edu.co

**Keywords:** water treatment, natural coagulants, turbidity removal, wastewater treatment, chemical-free treatment, sustainable water treatment, plant-based coagulants

## Abstract

This research evaluates extracts from the bark of *Heliocarpus popayanensis* and *Triumfetta bogotensis* as coagulating agents for removing turbidity in domestic wastewater, considering the coagulant dosage and pH of the wastewater. ANOVA was conducted to assess differences between the coagulants, dosages, and pH, with three pH levels (5, 8, and 9) and six dosages (7, 9, 11, 13, 15, and 17 mL per 1000 mL of wastewater) at a significance level of α = 0.05, and both the *p*-value and effect size were evaluated. This study found that the mucilaginous compound from the bark of *Triumfetta bogotensis* performed better in reducing turbidity levels, with an average reduction of 30.2 NTU (Nephelometric Turbidity Unit) (*CI* [25.9 NTU; 34.5 NTU], α = 0.05) at a pH of 5, and an average initial NTU of 102.2. This represents an average reduction of 70.45%. The dosage factor did not show significant effects on turbidity reduction, which opens the possibility for further study to determine the optimal dosage of the best coagulant.

## 1. Introduction

Synthetic flocculants are produced in large quantities by water clarification compound industries, as a process in which small particles coalesce into larger masses known as flocs with a lower density than water, allowing them to be removed. Originally, mineral-based flocculants such as calcium hydroxide, dolomitic lime, bentonite, clays, precipitated calcium carbonate, powdered activated carbon, and sand were used. However, synthetic and polymeric flocculants, such as polyacrylamides, polyamines, and synthetic electrolytes, are increasingly being introduced into the market due to their superior coagulation/flocculation performance. Nevertheless, they present important disadvantages such as a lack of sustainability according to some authors and potential health hazards to humans, including neurotoxicity and cancer [1,2,3,4].

An alternative to coagulation/flocculation in water treatment is the use of organic compounds, such as alginate extracted from laminaria seaweed, which is increasingly being used worldwide for drinking water treatment, as well as cellulose derivatives, certain vegetable starches, and gums [5,6].

In the treatment of wastewater and raw water, the stabilization of suspended particles is important, a process that is achieved through the addition of chemical compounds that allow the formation of conglomerates that subsequently precipitate [7]. Such a procedure is determined through a pilot test that is developed at the laboratory level, in which the optimal percentage of coagulant/flocculant substances that achieve the best removal efficiency of suspended particles that are the cause of high turbidity and unfit colors according to Colombian regulations for human consumption, is determined.

Coagulants and flocculants are chemical compounds that allow for reducing bacterial growth, the presence of algae, and the apparent color and turbidity of the water, due to the destabilization of the colloids that are suspended in it [8]. Colloids are stable suspensions that are impossible to separate without the addition of a coagulant, which allows the hydrophilic and hydrophobic particles of the colloid to separate due to their electrical charges.

After the chemical destabilization process that occurs when the coagulant is added, these particles agglomerate, forming a compact structure called flocs [7]. The formation of flocs is due to the breaking of van der Waals forces and the inactivation of electrostatic forces that prevent the agglomeration of the particles. For this reason, by adding a coagulant, electrostatic forces are blocked, allowing the particles to agglomerate and subsequently be removed from the water.

Coagulants/flocculants of metallic origin have been the most widely used for wastewater treatment. These include aluminum sulfate, ferric sulfate, ferrous sulfate, ferric chloride, and sodium aluminate [9].

Synthetic organic polymer coagulants and flocculants were developed based on polyelectrolytes and correspond to organic substances synthesized from nature. One of their main characteristics is the high molecular weight and net electrical charge. Organic polymers can be positively, negatively, or neutrally charged and, according to these charges, they function differently depending on the pH. For example, polyelectrolytes with positive electrical charge, also called cations, remove particles with negative electrical charge, but at low pH; those with negative electrical charge or anions remove particles with positive charge and at high pH; synthetic organic polymers with neutral charge can remove particles with positive or negative charge and at different pH ranges but require higher doses than with cationic or anionic polymers. Traditionally, these types of products are used as adjuvants to metal coagulants [9].

Natural coagulants and flocculants are an alternative source that natives have used in several countries of the world for water treatment. They are generated by spontaneous reactions produced in animals and plants, because of their metabolism or the biochemical reactions they produce [9]. An important aspect is that they have low toxicity and can be used as coagulating and flocculant agents, since they bind the particles suspended in the water, facilitating their sedimentation and subsequent removal.

When studying the biology and biochemistry of plants, the production of their own food (autotrophs) and the production of oxygen through photosynthesis are identified among their particularities. Plants have different structures and vegetative organs that allow their development, such as the roots, stems, leaves, flowers, fruits, and seeds, in which there are properties of vital importance for their industrial, commercial, and medicinal use.

In their transformation, plants produce a great number of chemical substances that have been employed in different uses. One of these substances is a polymer formed by glucose subunits—starch. According to [10,11], starch grains are carbohydrates, composed of two glucose subunits, called amylase and amylopectin, which plants synthesize from carbon dioxide (CO_2_). The content of both subunits depends on each plant, but amylopectin is between 70% and 80%, and amylase is between 20% and 30% in most plants.

Starch can be primary or secondary. Primary starch is stored in the chloroplasts of the plant and is used for energy assimilation during the photosynthesis process. Secondary starch is part of the energy reserve of plants. Both starches are found in tubers, bulbs, roots, and seeds, and have been used by mankind for thousands of years [11].

Among the relatively recent use of starch is its evaluation as a coagulant/flocculant. Researchers [12] have evaluated the use of cassava starch as a coadjuvant in the coagulation/flocculation process of domestic water. Among their most important findings is that the adequate concentration of aluminum sulfate and cassava starch, in a 1:3 ratio (aluminum sulfate/cassava starch), manages to reduce turbidity and apparent color by 75% and 78%, respectively. When using calcium hydroxide and cassava starch in a 1:1 ratio, the percentage of removal of both parameters was 35% and 67%, respectively. According to these results, it is evident that starch is an excellent raw material for water treatment and potabilization.

Starch from other plants has also been used for the preparation of coagulants/flocculants and adjuvants. According to [13], plantain starch has been shown to be a good coagulant, but with slow sedimentation. The authors of [14], on the other hand, have shown that potato starch presents turbidity removal values like those of aluminum sulfate, and higher values in the reduction in apparent color. However, its titration is recommended so as not to affect food availability.

Some traditional rural agro-industrial processes, such as “panela” production (a typical sweet in the gastronomy of several Central and South American countries obtained from sugarcane juice before undergoing the necessary purification process to obtain unrefined or brown sugar) [15], use coagulants for the juice clarification process, such as calcium hydroxide or phosphoric acid, and traditionally, plant-based flocculants [16,17,18] extracted from the bark of certain plants or trees in the form of cellulose or mucilage have also been used. Among these coagulants, the bark of the “Balso Blanco” tree (*Heliocarpus popayanensis*) (*Hp*) and the “Cadillo de Béstia” plant (*Triumfetta bogotensis*) (*Tb*) are mainly used, both of which are native species of Central America. *Hp* wood is mainly used for crafting, while the bark is an unusable byproduct, and *Tb* grows mainly in grasslands dedicated to livestock farming and is cut as a weed to avoid interfering with pasture growth and therefore livestock feed. These samples have been studied and determined according to the [19] classification system and are as follows:

*Heliocarpus popayanensis*:
Division   Magnoliophyta;KindMagnoliopsida;SubclassDilleniidae;OrderMalvales;FamilyTiliaceae;GenreHeliocarpus;SpeciesHeliocarpus Popayanensis.

*Triumfetta bogotensis DC*:
Division   Magnoliophyta;KindMagnoliopsida;SubclassDilleniidae;OrderMalvales;FamilyMalvaceae;Genre Triumfetta;SpeciesTriumfetta Bogotensis Dc.

The chemical characterization of the extracts used is summarized in the works from [20,21].

**Figure 1:**
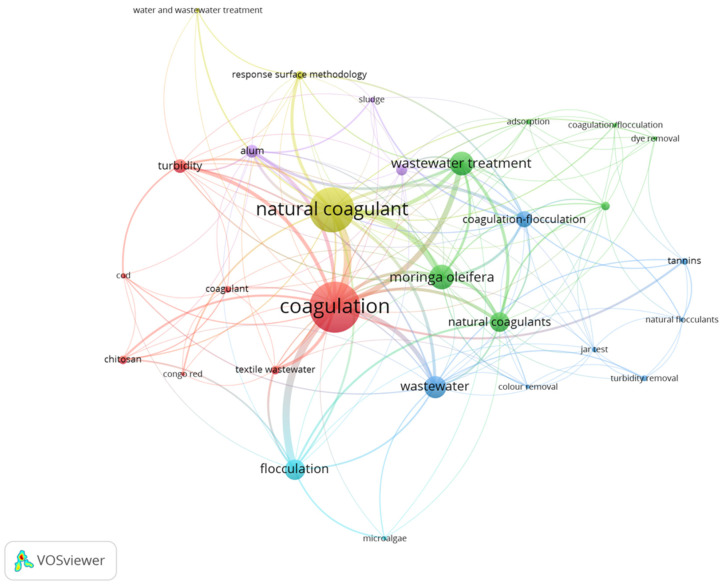
Author keyword co-occurrences in Scopus database for the keyword search “((“natural coagulant” OR “natural flocculant”) AND “wastewater”))” generated using VOSviewer [23].

Regarding the coagulation and removal of turbidity from plant-based compounds in water management, extracts from *Moringa oleifera* (*Mo*) are the most widely reported in the literature, as evidenced by Figure 1 generated using VOSviewer software v1.6.18 [22,23] from the search “(TITLE-ABS-KEY((“natural coagulant” OR “natural flocculant”) AND “wastewater”))” in the Scopus database.

*Moringa oleifera* (*Mo*) has been documented as a botanical specimen possessing attributes that enable the purification of water [5,24]. Presently, research efforts are underway to further investigate the properties of this plant [6,25] in the realm of wastewater treatment. Nevertheless, the manifold applications of this plant in the realm of nourishment underscore the exigency of exploring alternative natural solutions for water purification that do not compromise the integrity of food safety.

## 2. Methodology, Experimental Design, Data Measurement, and Analysis

### 2.1. Collection and Preparation of Plant Material

**Figure 2:**
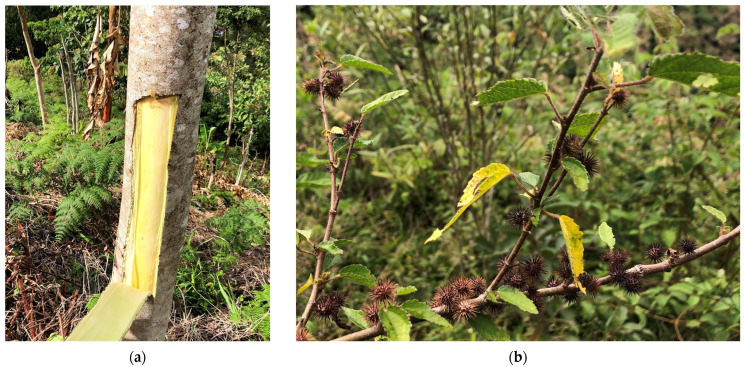
Plant material used: (**a**) the extraction of the bark from the *H. popayanensis*; (**b**) *Triumfetta bogotensis* plant found in pastures usually as an unutilized weed.

Traditionally, for the clarification of sugarcane juice, *Hp* extract is obtained from the bark of the tree through controlled cuts that allow the plant to recover in a few months (see Figure 2a). This bark is macerated to obtain a sticky liquid that is added to the sugarcane juice for the removal of turbidity, resulting in a compound called cachaza, which is used as animal feed or organic fertilizer for crops. The utilization of the *Tb* as a clarifier for sugarcane juices is relatively limited due to its diminutive size. Consequently, the extraction of its adhesive bark necessitates greater effort. Moreover, as it historically has been regarded as an undesired plant in pastures, it is often trimmed by operators in the livestock industry. The extraction process required for obtaining the extract to clarify sugarcane juice is destructive and necessitates a subsequent bark extraction process (see Figure 2b).

The wastewater samples were obtained from one of the rivers that crosses the city located in the study area, and the physicochemical characterization of the water is as follows:
Dissolved oxygen DO (mg/L):6.55 ± 0.3;BOD (mg/L):276 ± 28;COD (mg/L):440;pH:7.85;Conductivity (μs/cm):168.4;Turbidity (NTU):180;Total solids (mg/L):600;Total suspended solids (mg/L):   77 ± 30;Color (u Pt–Co):37;Temperature (°C):14.5;Flow rate (m^3^/s):1.4480.

### 2.2. Preparation of Plant Material to Obtain Coagulant Extract

For the extraction of mucilage, the methodology proposed in [16] was applied. Once the plant material was ground, 50 g of each plant was taken and separately liquefied with 200 mL of distilled water for approximately half a minute. The obtained mucilaginous material was packed in an amber glass bottle with 600 mL of distilled water, and then the material was separately shaken every 5 min, with the purpose of hydrating the extract to increase its viscosity. To separate the mucilaginous extract from the fiber of the plant material, it was filtered, 96% ethanol was then added in a 1:4 v/v ratio, the mixture was shaken, and finally the material was left to stand for precipitation.

For the best performance of the natural coagulant, physical–thermal hydrolysis was carried out at 60 °C during 1 h under constant agitation, in order to carry out the cleavage of the carbohydrates contained in the cellulose of the mucilaginous material, with the purpose of maximizing the coagulant properties of the extract.

### 2.3. Coagulation Study

For the preparation of the jar test, 1 L of wastewater sample was taken for each jar; the initial turbidity and pH then followed the recommendations of the Colombian Technical Standard NTC-3903 [26], initially with the fast speed “fast mixing” at 120 rpm; and once the doses of 7, 9, 11, 13, 15, and 19 mL per liter of water were added in the respective jars, 1 min of instantaneous mixing at constant speed was counted. The pH was selected from the range found in domestic wastewater in the city of Manizales and following the methodology proposed in [27].

After this, the slow mixing was started: the speed should be decreased until it is optimal to avoid breaking the flocs; this flocculation process was performed for 20 min at 40 rpm. Finally, the samples were left to rest for 15 min to achieve sedimentation of the flocs formed during the slow mixing. Once the time elapsed, a sample was taken from the broth to record the final turbidity [28]. A LaMotte 2020t/i turbidimeter (LaMotte Company, Chestertown, MD, USA, supplier: BIOWEB^®^ Colombia), adequately calibrated according to AMCO standards [29], was used to measure turbidity.

### 2.4. Organization and Loading of Data into the Software

**Figure 3:**
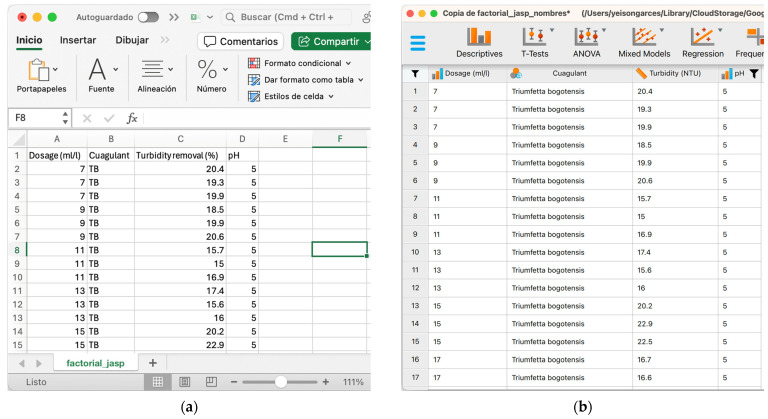
Organization of data in base form for data reading by the analysis software: (**a**) data sorted in Excel^®^, (**b**) data loaded into JASP software for analysis.

The collected and tabulated data were initially processed in Excel^®^ software v16.79 to generate a database that can be analyzed using JASP v0.17.3 [30] and rStudio v2023.09.0+463 (R software user interface [31]) software packages. The data should be organized in columnar form and saved in text file format (csv, txt, or similar), as shown in Figure 3a. Figure 3b illustrates the data loaded into the analysis software, where the Quagulant variable names or labels have been manipulated so that the results are displayed with the full names.

### 2.5. Analysis of Collected Data

The first analysis to be performed was the 2 × 3 × 6 factorial ANOVA (two different coagulants, 3 pH levels and 6 levels of coagulant dosage in milliliters per liter of water), which allows for determining the effects of multiple independent variables on a dependent variable in a cohort of subjects. In addition, factorial ANOVA allows, and requires, an assessment of whether there is an interaction between different levels of the independent variables [32]. Similarly, analysis of variance reports the effect size $\omega^{2}$, which is considered a good estimate when the sample size is small [33,34].

The result of the factorial ANOVA for the three factors is shown in Table 1, where it can be seen that each of the factors as well as their interactions are significant for turbidity removal (p<0.001); however, when analyzing the effect size $\omega^{2}$, it should be taken into account that the main effect sizes are centered on coagulant and pH (to analyze the effect size $\omega^{2}$ according to [33], the rule is that an effect size is trivial if $\omega^{2}<0.01$, small if it is between 0.01 and 0.06, medium between 0.06 and 0.14, and large if $\omega^{2}>0.14$).

**Figure 4:**
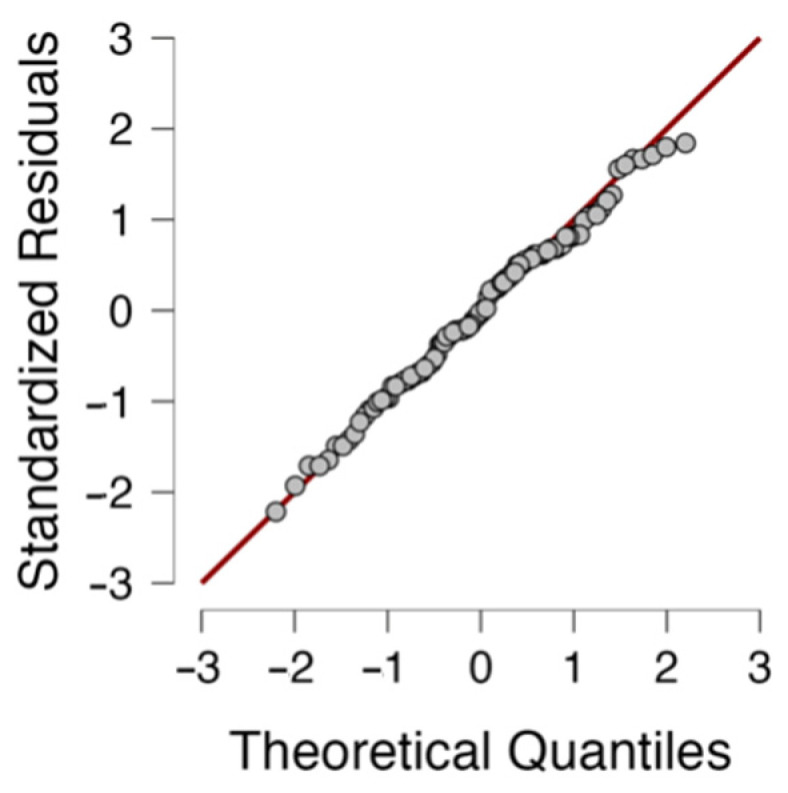
Q-Q plot for standardized residuals in the factorial ANOVA.

After generating the ANOVA table, it is very important in the analysis of variance to verify the assumptions of the model. There are two important assumptions to analyze: the assumption of equality of variances (homoscedasticity), which can be evaluated using Levene’s test, and the assumption of normality of the residuals, which can be evaluated using the Q-Q plot. For the case of the data analyzed, Levene’s test rejects the homoscedasticity assumption (f=2.236, p=0.002) with 95% confidence (α=0.05); the analysis consists of comparing the *p*-value of Levene’s test with the significance α, rejecting the hypothesis of equality of variances if p<α. Figure 4 implies the acceptance of the normality hypothesis since all the points are close to the unit line.

From the previous analysis, and after rejecting one of the assumptions, it is important to perform a non-parametric test. In the case of ANOVA, the Kruskal–Wallis test allows for verifying if the variation between the factors is significant for turbidity removal. Table 2 shows the results of the non-parametric test, where it is observed that the dosage factor is not significant for turbidity removal (p=0.432>α=0.05).

**Figure 5:**
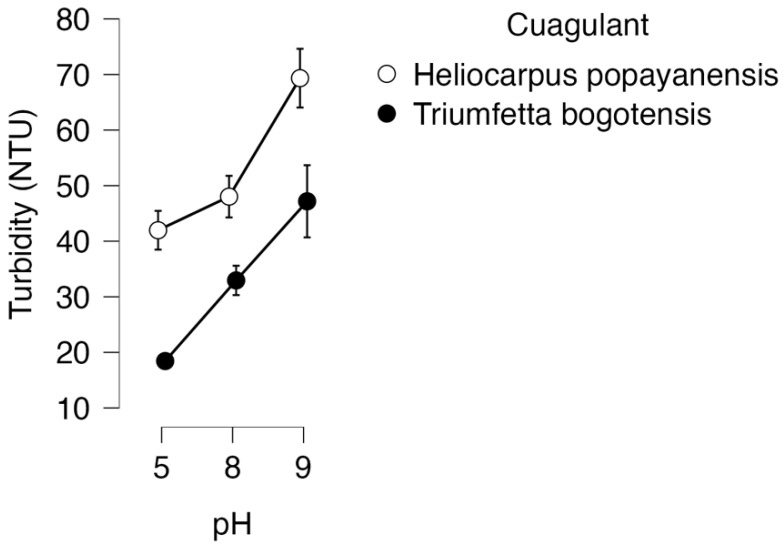
Descriptive graph of the performance of each coagulant at different pH.

With the numerical results complete, the graphical analysis allows for verifying the performance of the coagulant and pH factors. Figure 5 depicts the two coagulants and the three pH levels in relation to the efficiency of turbidity removal. It becomes apparent that the coagulant *Tb* exhibits a superior performance compared to *Hp*. Additionally, it is plausible to infer that a decrease in the pH of the wastewater leads to a more effective removal of turbidity.

**Figure 6:**
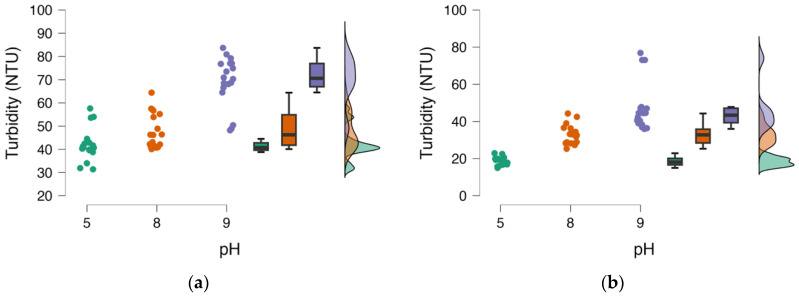
Distribution graphs of the data for each pH level for each coagulant: (**a**) *Heliocarpus popayanensis*, (**b**) *Triumfetta bogotensis*.

Figure 6 illustrates the distribution of the data for each coagulant independently at different pH levels. The analysis of this figure is interesting since it is possible to analyze how dispersed the data are, and the probability distribution of each variable can be easily visualized. It is interesting to analyze that for the case of the highest pH, in both coagulants, data far from the general group are observed; in the case of Hp, they are below; and in the case of Tb, they are higher. These data, besides moving the distribution away from the hypothesis of normality, could be considered as outlier data.

## 3. Conclusions

A methodology was developed to compare the performance of natural coagulants at different pH levels and coagulant concentrations. This methodology involved the organization of data, the processing through the JASP software, and the analysis of the results. Factors taken into consideration included the verification of assumptions and the type of tests necessary in cases of non-compliance. The analysis involved assessing the effect size and its corresponding reference values. Assumptions that were evaluated included the homogeneity of variances and normality of residuals for turbidity in NTU. If any of these assumptions were not met, the Kruskal–Wallis test was utilized, allowing for the elimination of the dosage factor from the analysis and indicating that it had no impact on turbidity removal. The effect size was evaluated using a specific statistic that, according to the literature, is deemed the most suitable for small samples.

The method required for the extraction of natural coagulants from two specific varieties of endemic plants that have traditionally been used in the clarification of sugar cane juice but have not been reported in the literature for their use in wastewater turbidity removal processes, was addressed.

Extracts derived from the bark of *Heliocarpus popayanensis* and *Triumfetta bogotensis* have demonstrated notable efficacy in eliminating turbidity from wastewater, particularly in instances where the pH levels are acidic. This methodology can be reproduced for various pH values and with alternative natural coagulant options that have been conventionally employed in the realm of food production. In similar works of coagulation/flocculation from plant extracts, there are reports of turbidity reduction similar to those reported in the present investigation of 70.45% (71–76% [25], 88,61% [2], 58–87% [4]), which suggests that the extracts of these two plant materials are promising in this area of study.

## Figures and Tables

**Table 1:** ANOVA for turbidity in (NTU).

Cases	Sum of Squares ^1^	df	Mean Square	F	*p*	ω^2^
Coagulant	11,077.763	1	11,077.763	3195.423	<0.001	0.334
pH	14,502.500	2	7251.250	2091.651	<0.001	0.437
Dosage (mL/L)	1615.651	5	323.130	93.208	<0.001	0.048
Coagulant × pH	371.315	2	185.657	53.554	<0.001	0.011
Coagulant × Dosage (mL/L)	1494.540	5	298.908	86.221	<0.001	0.045
pH × Dosage (mL/L)	2357.642	10	235.764	68.007	<0.001	0.070
Coagulant × pH × Dosage (mL/L)	1476.218	10	147.622	42.582	<0.001	0.043
Residuals	249.607	72	3.467			

^1^ Type III sum of squares.

**Table 2:** Kruskal–Wallis test for the factors.

Factor	Statistic	df	*p*
Coagulant	40.955	1	<0.001
pH	42.355	2	<0.001
Dosage (mL/L)	4.870	5	0.432

## Data Availability

The data presented in this study are available on request from the corresponding author.
